# Prognostic value of lymphatic vessel density in the capsule of early-stage hepatocellular carcinoma: implications for postoperative recurrence risk

**DOI:** 10.3389/fimmu.2026.1714314

**Published:** 2026-01-28

**Authors:** Jin Li, Yu-Bo Liang, Xing-Ming Chen, Zhi-Yan Ou, Qing-Bo Wang, Wan-Ling Luo, Yawhan Lakang, Zi-Sheng Yang, Jin-Xiang Zuo, Yu-Kai Li, Hao-Wen Qin, Xin-Wu Lv, Xiang Kui, Yan Wang, Yang Ke

**Affiliations:** 1 Department of Hepatobiliary Surgery, the Second Affiliated Hospital, Kunming Medical University, Kunming, Yunnan, China; 2 Department of Pathology, the Second Affiliated Hospital, Kunming Medical University, Kunming, Yunnan, China; 3 Department of International Healthcare, the First Affiliated Hospital, Kunming Medical University, Kunming, Yunnan, China; 4 Department of Surgical Education and Research, the Second Affiliated Hospital, Kunming Medical University, Kunming, Yunnan, China; 5 Yunnan Yunke Bio-Technology Institution, Kunming, Yunnan, China

**Keywords:** liver cancer, lymphangiogenesis, microvascular invasion, relapse, satellite nodules

## Abstract

**Objective:**

Lymphatic vessels are present in the capsule of hepatocellular carcinoma (HCC) at an early stage, but their value in the prognosis remains unclear. The study aimed to evaluate the prognostic impact of lymphatic vessels in the tumor capsule on patients with HCC at Barcelona Clinic Liver Cancer (BCLC) stages 0-A. This is one of the first studies to investigate the tumor capsule specifically.

**Methods:**

This retrospective study included HCC patients at BCLC stages 0-A, who underwent radical liver resection between January 2017 and December 2020. Lymphatic vessel density (LVD) in the tumor capsule was determined by immunohistochemistry using anti-D2-40. Patients were stratified into the high and low LVD groups. Their overall survival (OS) and recurrence-free survival (RFS) were analyzed. The potential risk factors affecting survival and for predicting microvascular invasion (MVI) or satellite nodules were analyzed using Cox regression analysis and logistic regression analysis, respectively.

**Results:**

A total of 212 patients were included (180 male and 142 patients < 60 years old). The 1, 3, and 5-year OS were 76.5%, 52.9%, and 41.2% in the high LVD group, versus 94.4%, 76.4%, and 69.0% in the low LVD group (*P* = 0.013). The 1, 3, and 5-year RFS were 30.6%, 30.6%, and 30.6% in the high LVD group, versus 72.5%, 52.2%, and 38.7% in the low LVD group (*P* = 0.014). High LVD in the tumor capsule was an independent risk factor for worse OS (HR = 2.145, 95% CI: 1.096-4.197, *P* = 0.026) and RFS (HR = 2.506, 95% CI: 1.197-5.243, *P* = 0.015), and also associated with the onset of MVI (OR = 8.493, 95% CI: 2.314-31.174, *P* = 0.001) and satellite nodules (OR = 5.755, 95% CI: 1.340-24.718, *P* = 0.019).

**Conclusions:**

High LVD in the tumor capsule was associated with worse OS, RFS, and intrahepatic spread (MVI and satellite nodules) in patients with HCC at BCLC stages 0-A after liver resection. Our findings suggest that assessing LVD in the tumor capsule could serve as a useful tool in predicting postoperative prognosis and guiding personalized treatment strategies for patients with early-stage HCC.

## Introduction

Radical liver resection is the mainstay therapy for patients with hepatocellular carcinoma (HCC) at the very early and early Barcelona Clinic Liver Cancer (BCLC 0-A) stages (1–3). Although the postoperative 5-year overall survival (OS) of patients with HCC at BCLC stages 0-A is above 75%, nearly half of these patients suffer from the postoperative recurrence within 5 years (4, 5), which is the main cause of their death (6–8). Hence, it is necessary to identify the risk factors of postoperative recurrence of HCC patients at the BCLC stages 0-A (9–11).

The tumor microenvironment (TME) of HCC is composed of immune cells, tumor-associated fibroblasts, endothelial cells, and other cell types, all of which play a crucial role in influencing tumor recurrence (12–15). The vessels that encapsulate tumor clusters (VETC) are a spider web-like vascular pattern encapsulating HCC, examined by immunostaining with anti-CD34 for classic blood vessels (16–18). The presence of VETC is associated with a high recurrence rate and poor OS in HCC patients at BCLC stages A-B following liver resection (19, 20). Additionally, VETC is valuable for predicting the response to conventional transarterial chemoembolization (cTACE), antiangiogenic therapies, and anti-PD-1 treatments in HCC patients (21–25). Therefore, the blood vessels in the tumor capsule play a critical role in the prognosis of HCC patients. However, the prognostic role of other vascular structures in the tumor capsule is rarely reported.

We have recently studied the role of tumor-associated lymphangiogenesis in the prognosis of cancer patients after radical resection (26, 27). We found that a high level of tumor-associated lymphatic vessel density (LVD) in the tumors was associated with worse OS and recurrence-free survival (RFS) in patients with HCC, cholangiocarcinoma, gallbladder cancer, and esophageal cancer after radical tumor resection (26, 27). Notably, in these studies, LVD was examined only within the tumor. However, lymphangiogenesis is mainly restricted in the tumor capsule of HCC (28). It is unknown whether LVD in the tumor capsule could predict outcomes of patients with HCC after radical liver resection.

The current retrospective study examined LVD in the tumor capsule and analyzed its prognostic values in patients with HCC at the early stage after radical liver resection. This study provides new insights into the determination of personalized and precise treatment strategies as well as post-operative monitoring in HCC patients (11, 29, 30).

## Materials and methods

This retrospective study was approved by the Committee of Ethics of the Second Affiliated Hospital of Kunming Medical University (Approval no. Shen-PJ-Ke-2024-300) and was performed following the Helsinki Declaration (31–33). All patients signed the informed consent form for the study.

### Patients

The inclusion criteria were (1) patients with newly diagnosed HCC at BCLC stages 0 or A underwent radical liver resection at the Second Affiliated Hospital of Kunming Medical University between January 2017 and December 2020; (2) HCC and R0 margin were confirmed by postoperative pathology. The exclusion criteria included (1) receiving any neoadjuvant treatment for HCC before liver resection, such as TACE, transcatheter arterial embolization, transcatheter arterial infusion, hepatic arterial infusion chemotherapy, targeted and/or immunotherapy; (2) simultaneously diagnosed with other malignant tumors or life-threatening diseases; (3) incomplete clinical, pathological, or survival data.

### Data collection

The demographic and clinical data of individual patients were collected from the medical record system of the Second Affiliated Hospital of Kunming Medical University. The demographic parameters included age, which was divided into < 60 years old or ≥ 60 years old (34); sex (35). The clinical data contained preoperative laboratory parameters, such as albumin-bilirubin (ALBI) grade, which was an alternative indicator to the Child-Pugh score specifically in patients with HCC and was divided into 1, 2, or 3 grades (36–39); hepatitis B and/or C viral infection (40); and serum α-fetoprotein (AFP) levels, which were divided into < 400 ng/mL or ≥ 400 ng/mL (41–44). Preoperative radiological parameters included liver cirrhosis (45–48); maximum tumor diameter, which was divided into < 2 cm or ≥ 2 cm (49, 50); and the number of tumor nodules, which was divided into single nodule or multiple nodules (51, 52).

### Pathological evaluation of microvascular invasion and satellite nodules

The surgical liver specimens were serially sectioned with an interval of 10 mm and fixed in 20% formalin, followed by paraffin-embedded. The tissue sections (4 μm in thickness) were stained with Hematoxylin and Eosin. Microvascular invasion (MVI) was present as a cluster of cancer cells in the microvascular lumen (53). Satellite nodules were identified as small cancerous foci located in the liver parenchyma within 2 cm distance from the main tumor, and were separated from the main tumor by non-tumor liver tissue (54).

### Pathological evaluation of tumor capsule lymphatic vessel density

The density of tumor capsule lymphatic vessels was semi-quantified by immunochemistry using anti-D2-40 (55, 56). Briefly, the formalin-fixed and paraffin-embedded tumor sections (4 μm in thickness) were dewaxed, rehydrated and treated with 0.3% H_2_O_2_ in methanol to quench endogen peroxidase. The sections were subjected to antigen retrieval in 0.1 mol/l citrate buffer (pH 5.5) at 100°C for 60 min. After being washed, the tissue sections were incubated with the monoclonal anti-D2-40 (CDM-0010, Celnovte, Zhengzhou, Henan, China) for 60 min at 4°C. The bound antibody was detected with Microstacker™Rx Poly-HRP Conjugated Secondary Antibody Polymer (SD3102, Celnovte) and visualized with DBA (SD5600, Celnovte) followed by counterstaining with hematoxylin.

To quantify LVD in the tumor capsule, three areas of the highest vascular density, *a.k.a.* vascular hot spots, in the tumor capsule of HCC were identified at 40 × magnification, then the numbers of lymphatic vessels in these three vascular hot spots were manually counted at 200 × magnification by two independent investigators (H.W.Q. and X.W.L.) in a blinded manner, and discrepancies were evaluated by Y.W. (8, 9, and > 20 years of experience in pathology, respectively). A final LVD value was obtained from the average of the six counts (there were two investigators, and each investigator counted the three vascular hot spots independently).

### Grouping

The optimal cut-off value for LVD was determined using X-tile software (https://medicine.yale.edu/lab/rimm/research/software/, Yale School of Medicine, New Haven, CT, USA) (57, 58). Continuous LVD values, OS time, and survival status for all patients were input into the program. The “Kaplan-Meier” module was selected, and the log-rank test was applied as the optimization criterion. The cut-off value for LVD that resulted in the most significant survival difference between groups was chosen. Based on this, patients were stratified into the ‘‘high LVD’’ and ‘‘low LVD’’ groups.

### Follow-up

Those patients were followed up, every 1–2 months during the first 6 months post-surgery, every 3–4 months thereafter until 2 years post-surgery, and every 5–6 months thereafter. During the follow-up, individual patients underwent physical examinations, liver function tests, tumor marker tests, chest X-ray or CT, and at least one abdominal imaging, including liver ultrasound, triphasic liver CT, or MRI at each follow-up. Tumor recurrence was diagnosed by imaging evidence of HCC on liver ultrasound, triphasic liver CT, or MRI, and one with recurrence was treated with a liver transplantation, liver resection, radio-frequency ablation, TACE, systemic therapy, or best supportive care as appropriate (59, 60). The RFS was determined from the date of liver resection to the date of recurrence or death. The OS was determined from the date of liver resection to the date of death from any cause (61). Follow-up was conducted until January 2025, which was defined as the last follow-up date.

### Sample size

The required sample size was calculated using G*Power software (version 3.1.9.7; Franz Faul, Universität Kiel, Kiel, Germany) (62). Based on a previous systematic review (26), we estimated that a higher level of LVD in the tumor capsule was significantly associated with worse OS in HCC patients (HR = 2.35). We calculated that at least 189 patients would be required in order to detect this expected difference with 95% power at a 5% significance level.

### Statistical analysis

Data were analyzed using SPSS 26.0 (SPSS, Chicago, IL, USA), X-tile software (https://medicine.yale.edu/lab/rimm/research/software), and the logistf package in R-4.3.1 (https://cran.r-project.org/bin/windows/base/old/). Cohen’s kappa coefficient was used to assess inter-observer agreement for the categorization of LVD. Categorical variables were presented as numbers and percentages, and they were analyzed by the chi-square test, Fisher’s exact test, or the Mann-Whitney U test as appropriate.

Survival curves were generated using the Kaplan-Meier method and tested by the log-rank test. The potential risk factors affecting their survival were first analyzed using univariate Cox regression. Factors with a P-value < 0.05 in the univariate analysis were then included in the multivariate regression analysis, using backward likelihood ratio selection.

Univariate logistic regression was performed to identify risk factors of MVI and satellite nodules. When rare cases occurred in binary logistic regression, Firth’s bias-reduced penalized-likelihood logistic regression, implemented using the logistf package in R-4.3.1, was used to adjust for rare event data. Factors with a P-value < 0.05 were included in the multivariate logistic regression analysis using backward likelihood ratio selection. A difference was considered significant if the P-value was < 0.05.

## Results

### Patient characteristics

**Figure 1:**
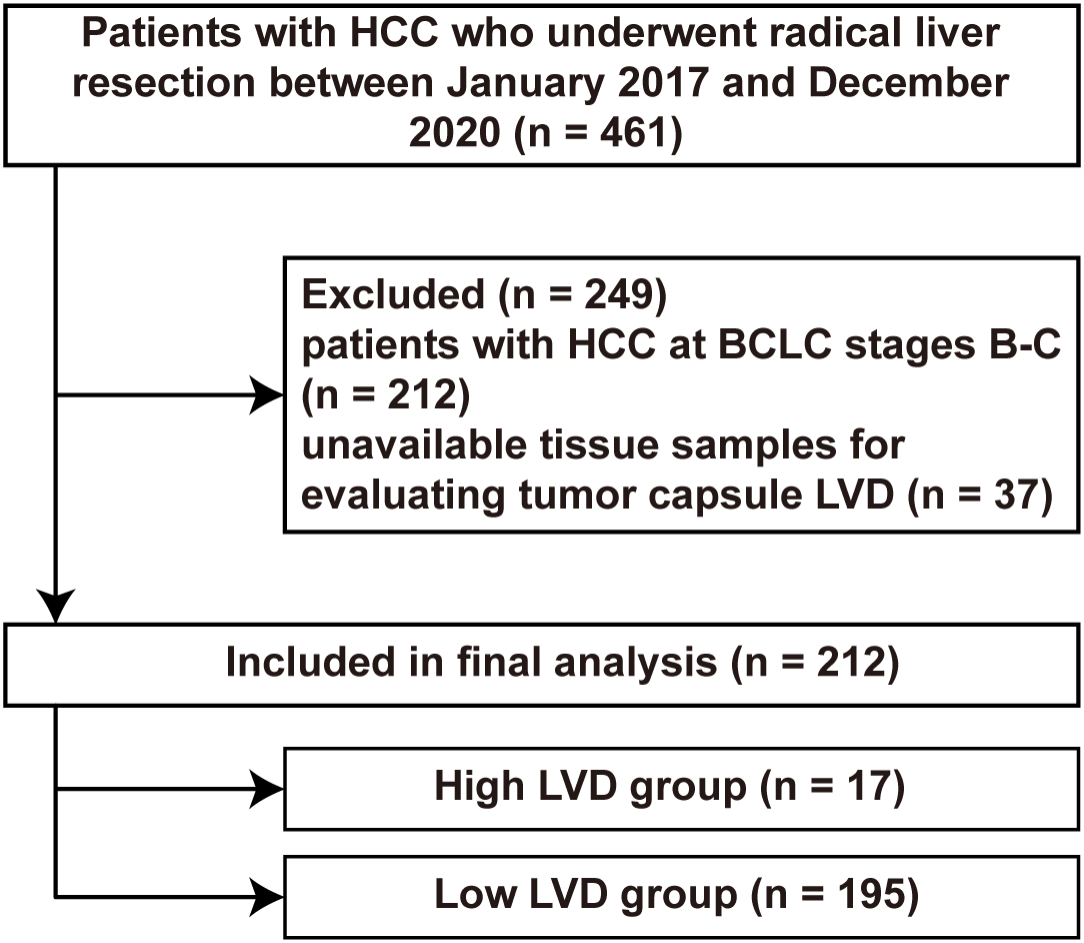
A flowchart of patient selection. BCLC, Barcelona Clinic Liver Cancer stage; HCC, hepatocellular carcinoma; LVD, lymphatic vessel density.

A total of 461 HCC patients consecutively underwent radical liver resection at the Second Affiliated Hospital of Kunming Medical University between January 2017 and December 2020 and were retrospectively screened (**Figure 1**). Among them, 249 patients were excluded due to the following reasons: 212 patients with HCC at BCLC stages B-C; 37 patients without available tissue samples for evaluating LVD. Finally, the remaining 212 patients were included for this retrospective analysis.

Their demographic and clinical characteristics are shown in **Table 1**. Of them, 180 (84.9%) patients were male, and 142 (67.0%) were under 60 years old. The ALBI grade was 1 in 86 (40.6%) patients, 2 in 66 (31.1%) patients, and 3 in 60 (28.3%) patients. There were 162 (76.4%) patients with HBV and/or HCV infection, and 141 (66.5%) patients with liver cirrhosis. There were 44 (20.8%) patients with a serum AFP level ≥ 400 ng/mL. There were 202 (95.3%) patients with a maximum tumor diameter of ≥ 2 cm, and 205 (96.7%) HCCs with a single nodule.

**Table 1:** Association of LVD in the tumor capsule with demographic and preoperative clinical characteristics of patients with hepatocellular carcinoma at Barcelona Clinic Liver Cancer stages 0-A.

Variables	Total (n = 212)	High LVD group (n = 17)	Low LVD group (n = 195)	P-value
Sex				0.451
Male	180 (84.9%)	16 (94.1%)	164 (84.1%)	
Female	32 (15.1%)	1 (5.9%)	31 (15.9%)	
Age (years)				0.742
≥ 60	70 (33.0%)	5 (29.4%)	65 (33.3%)	
< 60	142 (67.0%)	12 (70.6%)	130 (66.7%)	
ALBI grade				0.795
1	86 (40.6%)	7 (41.2%)	79 (40.5%)	
2	66 (31.1%)	6 (35.3%)	60 (30.8%)	
3	60 (28.3%)	4 (23.5%)	56 (28.7%)	
HBV and/or HCV infection				1.000
Yes	162 (76.4%)	13 (76.5%)	149 (76.4%)	
No	50 (23.6%)	4 (23.5%)	46 (23.6%)	
AFP (ng/mL)				0.357
≥ 400	44 (20.7%)	5 (29.4%)	39 (20.0%)	
< 400	168 (79.3%)	12 (70.6%)	156 (80.0%)	
Cirrhosis				0.149
Yes	141 (66.5%)	14 (82.4%)	127 (65.1%)	
No	71 (33.5%)	3 (17.6%)	68 (34.9%)	
Maximum tumor diameter (cm)				1.000
≥ 2	202 (95.3%)	17 (100.0%)	185 (94.9%)	
< 2	10 (4.7%)	0 (0.0%)	10 (5.1%)	
Single nodule				1.000
Yes	205 (96.7%)	17 (100.0%)	188 (96.4%)	
No	7 (3.3%)	0 (0.0%)	7 (3.6%)	

ALBI, albumin and total bilirubin; AFP, alpha fetoprotein; HBV, hepatitis B virus; HCV, hepatitis C virus; LVD, lymphatic vessel density.

**Figure 2:**
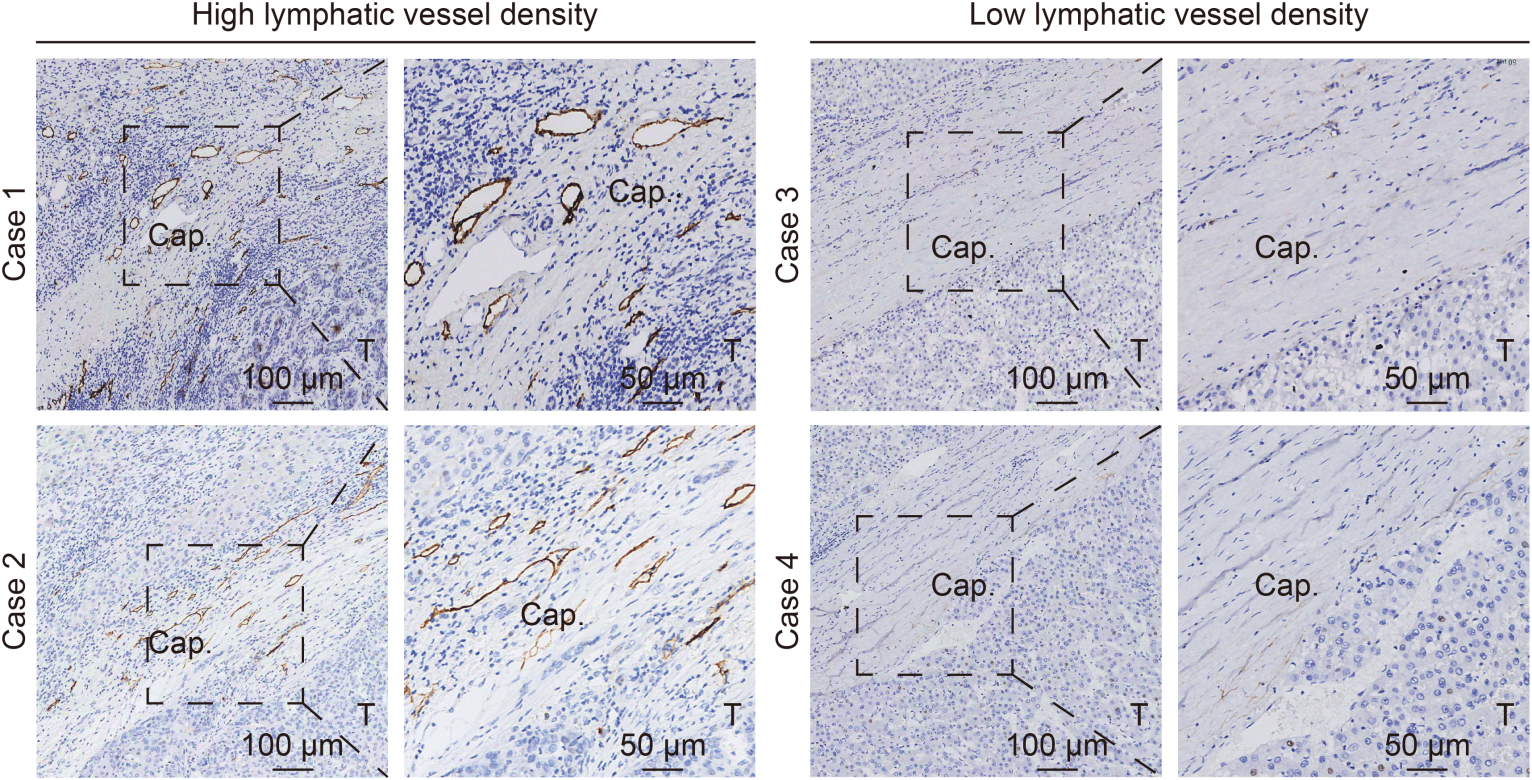
Immunohistochemical images of representative cases of groups with high and low lymphatic vessel density in the tumor capsule of hepatocellular carcinoma. Lymphatic vessels were stained using anti-D2–40 antibody. Cap., tumor capsule; T., tumor.

Analyses of LVD in the tumor capsule using X-tile revealed that its cut-off value was 13.0. Among those 212 patients, 195 (92.0%) patients were stratified into the “low LVD” group, and 17 (8.0%) patients were allocated to the “high LVD” group (**Figure 1**, **Table 2**). There was no significant difference in the demographic data, clinical ALBI grade, hepatitis virus infection, AFP levels, liver cirrhosis, maximum tumor diameter, or tumor number between those two groups of patients (all *P* > 0.05). The representative HCC cases with high and low LVD are shown in **Figure 2**.

**Table 2:** Cox regression analysis of risk factors for overall survival in patients with hepatocellular carcinoma at Barcelona Clinic Liver Cancer stages 0-A.

Variables	Univariate analysis			Multivariate analysis		
	P-value	HR	95% CI	P-value	HR	95% CI
Sex (male vs. female)	0.540	1.231	0.633-2.395			
Age (≥ 60 years vs. < 60 years)	0.812	1.059	0.659-1.702			
HBV and/or HCV (yes vs. no)	0.083	0.648	0.397-1.058			
AFP (≥ 400 ng/mL vs. < 400 ng/mL)	0.021	1.791	1.090-2.942	0.021	1.802	1.095-2.965
ALBI (2 vs. 1)	0.903	0.969	0.581-1.614			
ALBI (3 vs. 1)	0.116	0.621	0.343-1.125			
Cirrhosis (yes vs. no)	0.003	2.333	1.326-4.105	0.006	2.211	1.254-3.899
Max tumor diameter (≥ 2 cm vs. < 2 cm)	0.314	1.810	0.570-5.743			
Single tumor (yes vs. no)	0.575	0.669	0.164-2.727			
LVD (high vs. low)	0.016	2.264	1.163-4.408	0.026	2.145	1.096-4.197

ALBI, albumin and total bilirubin; AFP, alpha fetoprotein; CI, confidential interval; HBV, hepatitis B virus; HCV, hepatitis C virus; HR, hazard ratio; LVD, lymphatic vessel density.

Cohen’s kappa coefficient was 0.81 for the categorization of LVD.

### Effect of LVD in the tumor capsule on OS and RFS

**Figure 3:**
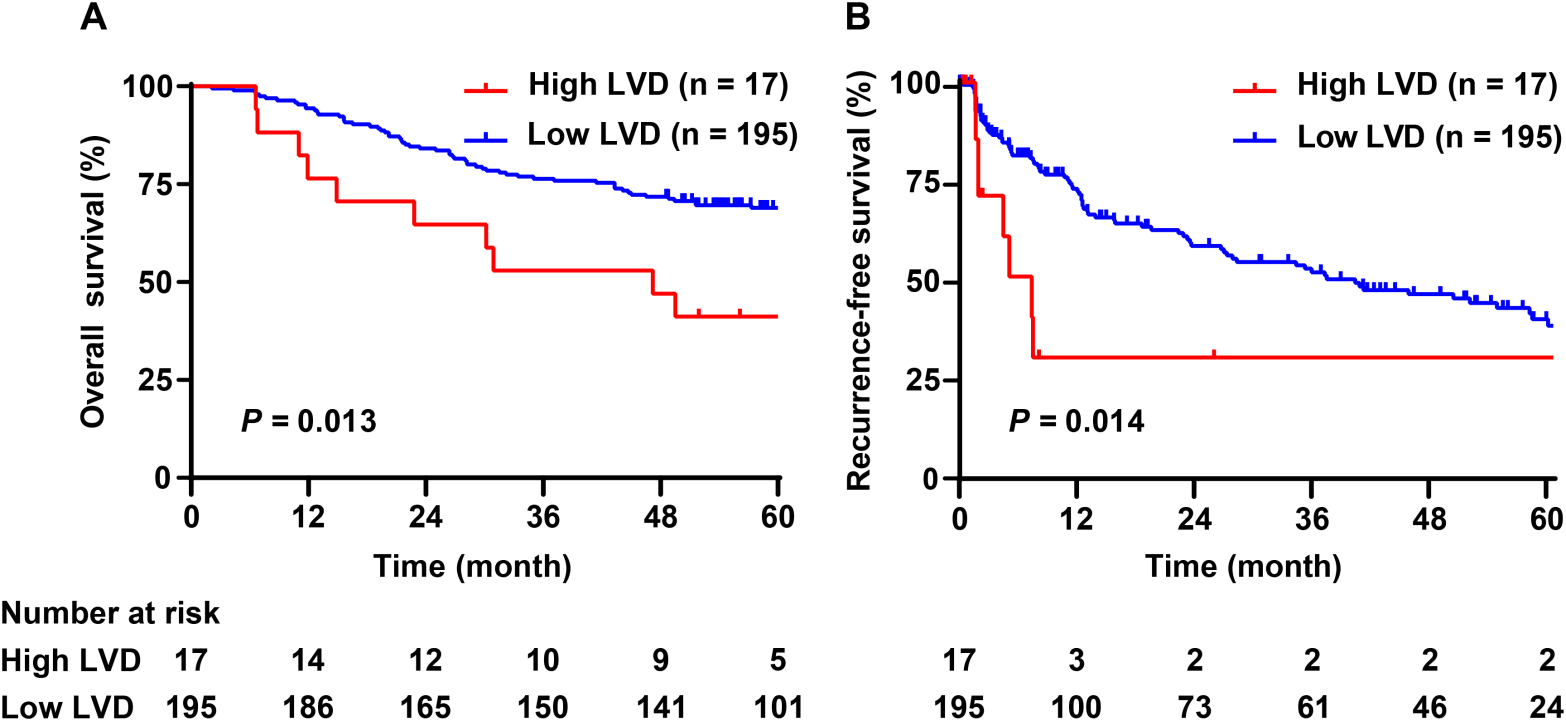
Comparisons of survival of patients with hepatocellular carcinoma at Barcelona Clinic Liver Cancer stages 0-A with high or low LVD in the tumor capsule. **(A)**, overall survival; **(B)**, recurrence-free survival. LVD, lymphatic vessel density.

The median follow-up was 68.9 (95% CI: 64.8-72.5) months. Among the 212 patients, 76 (35.8%) patients died. The median OS was 47.2 (95% CI: 21.2-73.2) months in the high LVD group, versus over 60.0 months in the low LVD group, respectively (*P* = 0.013, **Figure 3A**). The postoperative OS at 1, 3, and 5 years was 76.5%, 52.9%, and 41.2% in the high LVD group and 94.4%, 76.4%, and 69.0% in the low LVD group, respectively.

During the follow-up period, 92 (43.3%) patients experienced HCC recurrence. The median RFS was 7.3 (95% CI: 3.1-11.4) months in the high LVD group, versus 40.0 (95% CI: 21.8-58.2) months in the low LVD group, respectively (*P* = 0.014, **Figure 3B**). The postoperative RFS at 1, 3, and 5 years was 30.6%, 30.6%, and 30.6% in the high LVD group and 72.5%, 52.2%, and 38.7% in the low LVD group, respectively.

### Independent risk factors for OS and RFS

Univariate Cox regression analysis for OS indicated that the levels of AFP ≥ 400 ng/mL (HR = 1.791, 95% CI 1.090-2.942, *P* = 0.021, **Table 2**), cirrhosis (HR = 2.333, 95% CI 1.326-4.105, *P* = 0.003), and a high level of LVD in the tumor capsule (HR = 2.264, 95% CI 1.163-4.408, *P* = 0.016) were significantly associated with a worse OS in this population. Multivariate Cox regression analysis revealed that the levels of AFP ≥ 400 ng/mL (HR = 1.802, 95% CI 1.095-2.965, *P* = 0.021), cirrhosis (HR = 2.211, 95% CI 1.254-3.899, *P* = 0.006), and a high level of LVD in the tumor capsule (HR = 2.145, 95% CI 1.096-4.197, *P* = 0.026) were independent risk factors for a worse OS in this population.

Univariate Cox regression analysis for RFS unveiled that cirrhosis (HR = 1.592, 95% CI 1.009-2.510, *P* = 0.046, **Table 3**) and a high level of LVD in the tumor capsule (HR = 2.443, 95% CI 1.169-5.104, *P* = 0.018) were significantly associated with a worse RFS. Multivariate Cox regression analysis uncovered that cirrhosis (HR = 1.612, 95% CI 1.021-2.546, *P* = 0.040) and a high level of LVD in the tumor capsule (HR = 2.506, 95% CI 1.197-5.243, *P* = 0.015) were independent risk factors for a worse RFS in this population.

**Table 3:** Cox regression analysis of risk factors for recurrence-free survival in patients with hepatocellular carcinoma at Barcelona Clinic Liver Cancer stages 0-A.

Variables	Univariate analysis			Multivariate analysis		
	P-value	HR	95% CI	P-value	HR	95% CI
Sex (male vs. female)	0.080	1.853	0.929-3.695			
Age (≥ 60 years vs. < 60 years)	0.505	1.162	0.748-1.805			
HBV and/or HCV (yes vs. no)	0.547	0.861	0.528-1.402			
AFP (≥ 400 ng/mL vs. < 400 ng/mL)	0.076	1.556	0.954-2.538			
ALBI (2 vs. 1)	0.408	0.812	0.496-1.330			
ALBI (3 vs. 1)	0.754	0.923	0.559-1.523			
Cirrhosis (yes vs. no)	0.046	1.592	1.009-2.510	0.040	1.612	1.021-2.546
Max tumor diameter (≥ 2 cm vs. < 2 cm)	0.299	1.842	0.582-5.827			
Single tumor (yes vs. no)	0.443	1.481	0.543-4.044			
LVD (high vs. low)	0.018	2.443	1.169-5.104	0.015	2.506	1.197-5.243

ALBI, albumin and total bilirubin; AFP, alpha fetoprotein; CI, confidential interval; HBV, hepatitis B virus; HCV, hepatitis C virus; HR, hazard ratio; LVD, lymphatic vessel density.

### Independent risk factors for MVI

**Figure 4:**
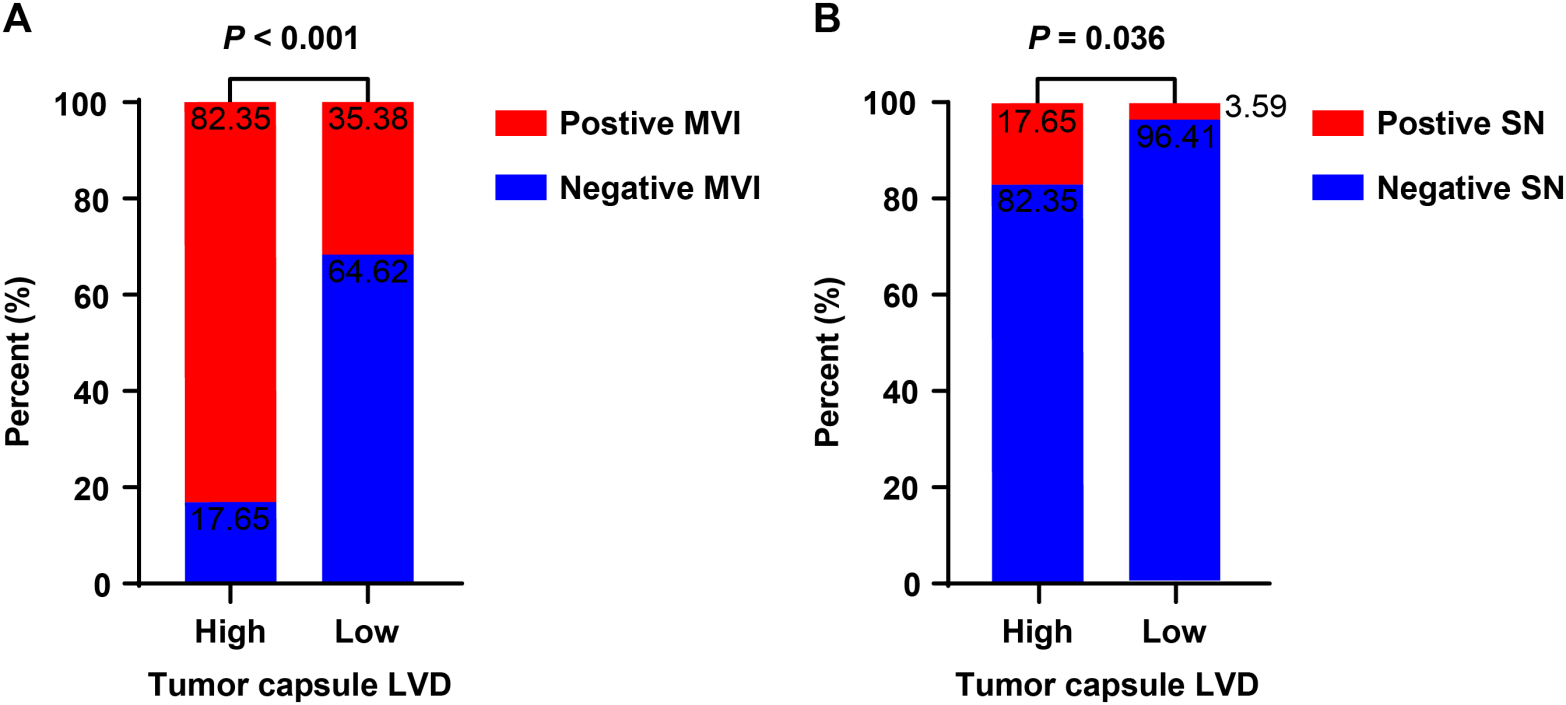
The association between LVD and MVI or satellite nodules in hepatocellular carcinoma at Barcelona Clinic Liver Cancer stages 0-A. **(A)**, MVI; **(B)**, satellite nodules. LVD, lymphatic vessel density; MVI, microvascular invasion; SN, satellite nodules.

Histological examinations exhibited that 83 (39.2%) HCC patients displayed positive MVI. The positive rate of MVI was 82.4% in the high LVD group of patients and significantly higher than 35.4% in the low LVD group (*P* < 0.001, **Figure 4A**). Further univariate logistic regression analysis indicated that the levels of AFP ≥ 400 ng/mL (OR = 3.176, 95% CI 1.599-6.310, *P* < 0.001) and a high level of LVD in the tumor capsule (OR = 8.522, 95% CI 2.367-30.681, *P* = 0.001) were significantly associated with the development of positive MVI (**Table 4**). Multivariate logistic regression analysis revealed that the levels of AFP ≥ 400 ng/mL (OR = 3.168, 95% CI 1.563-6.423, *P* < 0.001) and a high level of LVD in the tumor capsule (OR = 8.493, 95% CI 2.314 - 31.174, *P* = 0.001) were independent risk factors for the presence of MVI.

**Table 4:** Logistic regression analysis of risk factors for microvascular invasion in hepatocellular carcinoma at Barcelona Clinic Liver Cancer stages 0-A.

Variables	Univariate analysis			Multivariate analysis		
	P-value	OR	95% CI	P-value	OR	95% CI
Sex (male vs. female)	0.323	1.501	0.671-3.356			
Age (≥ 60 years vs. < 60 years)	0.472	0.805	0.445-1.455			
HBV and/or HCV (yes vs. no)	0.545	0.818	0.428-1.565			
AFP (≥ 400 ng/m vs. < 400 ng/m)	< 0.001	3.176	1.599-6.310	< 0.001	3.168	1.563-6.423
6.423ALBI (2 vs. 1)	0.783	1.097	0.567-2.121			
ALBI (3 vs. 1)	0.587	1.205	0.614-2.366			
Cirrhosis (yes vs. no)	0.154	1.546	0.849-2.813			
Max tumor diameter (≥ 2 cm vs. < 2 cm)	0.231	2.241	0.598-8.397			
Single tumor (yes vs. no)	0.203	0.250	0.030-2.115			
LVD (high vs. low)	0.001	8.522	2.367-30.681	0.001	8.493	2.314-31.174

ALBI, albumin and total bilirubin; AFP, alpha fetoprotein; CI, confidential interval; HBV, hepatitis B virus; HCV, hepatitis C virus; LVD, lymphatic vessel density; OR, odds ratio.

### Independent risk factors for satellite nodules

There were 10 (4.7%) HCC patients with positive satellite nodules. The positive rate of satellite nodules was 17.7% in the high LVD group of patients and significantly higher than 3.6% in the low LVD group (*P* = 0.036, **Figure 4B**). Univariate logistic regression analysis exhibited that a high level of LVD in the tumor capsule was significantly associated with the presence of satellite nodules (OR = 5.755, 95% CI 1.340-24.718, *P* = 0.019, **Table 5**). Further multivariate logistic regression analysis displayed that a high level of LVD in the tumor capsule was identified as the only independent risk factor for the development of satellite nodules in this population of HCC (OR = 5.755, 95% CI 1.340-24.718, *P* = 0.019).

**Table 5:** Logistic regression analysis of risk factors for satellite nodules in hepatocellular carcinoma at Barcelona Clinic Liver Cancer stages 0-A.

Variables	Univariate analysis			Multivariate analysis		
	P-value	OR	95% CI	P-value	OR	95% CI
Sex (male vs. female)	0.241	4.003	0.493-519.480			
Age (≥ 60 year vs. < 60 year)	0.835	0.864	0.216-3.446			
HBV and/or HCV (yes vs. no)	0.206	0.430	0.116-1.590			
AFP (≥ 400 ng/m vs. < 400 ng/m)	0.138	2.700	0.727-10.023			
ALBI (2 vs. 1)	0.741	1.317	0.257-6.748			
ALBI (3 vs. 1)	0.384	1.976	0.426-9.171			
Cirrhosis (yes vs. no)	0.364	2.075	0.429-10.040			
Max tumor diameter (≥ 2 cm vs. < 2 cm)	0.772	1.496	0.174-196.180			
Single tumor (yes vs. no)	0.888	1.241	0.009-11.472			
LVD (high vs. low)	0.019	5.755	1.340-24.718	0.019	5.755	1.340-24.718

ALBI, albumin and total bilirubin; AFP, alpha fetoprotein; CI, confidential interval; HBV, hepatitis B virus; HCV, hepatitis C virus; LVD, lymphatic vessel density; OR, odds ratio.

## Discussion

Patients with HCC at BCLC stages 0-A who underwent radical liver resection can achieve a 5-year OS of over 75% but nearly half of these patients suffer from HCC recurrence within 5 years after operation (4). A high density of blood vessels in tumor capsule has been predicted to have a worse survival and tumor recurrence after liver resection in patients with BCLC stage A (19). However, the prognostic role of other structures in the tumor capsule is rarely explored. Given that lymphatic vessels are present in the capsule of HCC at an early stage (28, 63), this study investigated the predictive value of LVD in the tumor capsule in the OS and RFS of patients with HCC at BCLC stages 0-A after radical liver resection.

We were among the first to find that a high level of LVD in the tumor capsule was associated with shorter OS and RFS of patients with HCC at BCLC stages 0-A who underwent radical liver resection, compared to those with a low level of LVD in the tumor capsule. A high level of LVD in the tumor capsule was an independent risk factor for a worse postoperative OS and RFS. These findings were partly consistent with a previous observation that a high level of LVD within HCC acts as an independent risk factor for worse RFS, but only marginal significance for OS (64). The difference may stem from the fact that our study centered exclusively on patients with HCC at BCLC stages 0-A, while their study included patients with HCC at BCLC stages 0-C (64). The worse survival of patients with HCC at BCLC stages B-C may mask the prognostic impact of lymphatic vessels in their study. Furthermore, while we measured the LVD in the capsule of HCC, they analyzed the LVD in the tumor interior. These support the notion that the margin of HCC may have more significant malignant activity than its interior (65, 66). Conceivably, analysis of LVD in the tumor capsule may be valuable for evaluating its prognostic value in the survival of HCC patients.

This study was among the first to find the significant association between LVD in the tumor capsule and the presence of MVI, as well as LVD in the tumor capsule and the development of satellite nodules in HCC. A high level of LVD in the tumor capsule was an independent risk factor of the presence of MVI (OR = 8.493, P = 0.001) and the development of satellite nodules (OR = 5.755, P = 0.019) in patients with HCC at BCLC stages 0-A. Notably, the presence of lymphatic tumor infiltration is a high-risk factor of HCC recurrence (64), suggesting that measurement of lymphatic vessels in the tumor capsule may be a better strategy to predict HCC recurrence and patients’ survival.

Satellite nodules arise from MVI (67). Previous studies have shown that the formation of MVI and satellite nodules in the liver occurs primarily through small blood vessels (68), while lymphatic vessels in HCC are mainly associated with lymphatic node metastasis, a hallmark of BCLC stage C (69). Our results for the first time indicated that in the very early and early stages of HCC, lymphatic vessels in the tumor capsule could also promote tumor spread to form these intrahepatic microlesions. Given that the rate of lymphatic node metastasis is low (3.3%) in HCC (70), the role of lymphatic vessels in promoting intrahepatic spread seems to be more dangerous and warrants more attention compared to its role in promoting lymphatic node metastasis. Moreover, the MVI and satellite nodules are widely accepted as high-risk factors for the recurrence of HCC after liver resection, which supports the association of LVD in the tumor capsule with the prognosis of patients with HCC at BCLC stages 0-A.

Why does lymphangiogenesis in the tumor capsule promote HCC progression? As far as we know, firstly, lymphatic vessels function as a direct channel for tumor cell spread (71–73). The entry of tumor cells into lymphatic vessels was termed lymphatic vascular invasion (74). HCC patients with positive lymphatic vascular invasion usually have a shorter RFS and OS compared to those without lymphatic vascular invasion (75). Secondly, lymphatic endothelial cells may impair anti-tumor immunity, thus indirectly contributing to tumor progression (76, 77). Thirdly, lymphatic endothelial cells may directly communicate with tumor cells to stimulate tumor cell growth and metastasis. For example, lymphatic endothelial cells can secrete CCL21, which binds to the CCR7 receptor on tumor stem cells to stimulate their growth (78). We are interested in further validating whether these mechanisms observed in other tumors could also occur in HCC.

The results of the current study should be interpreted in the context of certain limitations. Firstly, the retrospective design and the single-center nature may potentially affect data reproducibility. Secondly, the sample size was relatively smaller, especially only 17 patients with a high level of LVD in the tumor capsule, which may limit the statistical power to identify significant associations. Thirdly, there was no standardized cutoff value for high and low levels of LVD in the tumor capsule in HCC worldwide. The cutoff value of LVD in the tumor capsule in this study should be further validated.

## Conclusions

High level of LVD in the tumor capsule is associated with worse OS, RFS, and intrahepatic spread (MVI and satellite nodules) in patients with HCC at BCLC stages 0-A after radical liver resection. Assessing LVD in the tumor capsule may help identify patients at high risk of HCC recurrence, thereby assisting medical centers in optimizing the allocation of limited clinical resources.

## Data Availability

The raw data supporting the conclusions of this article will be made available by the authors, without undue reservation.
